# The benefit of cooperation evaluation among hospitals in department of health and welfare of Muhammadiyah organization

**DOI:** 10.1186/1471-2458-14-S1-O6

**Published:** 2014-01-29

**Authors:** Muhammad Natsir Nugroho, Laksono Trisnantoro, Eddy Purnomo

**Affiliations:** 1 Islamic Hospital Pondok Kopi, Jakarta Timur 13460, Indonesia; 2 Universitas Gadjah Mada, Yogyakarta 55281, Indonesia

## Background

Preparing to compete in years to come, Muhammadiyah hospitals in Indonesia urge to have a reliable integrated management system. This study presents an evaluation result in terms of integrated management system from several Muhammadiyah hospitals in East Java and Central Java. The objective was to choose which system would be the best integrated management system to be applied in all Muhamadiyah Hospitals in Indonesia.

## Materials and methods

A qualitative and quantitative method (mixed method) approach was used in this research. The qualitative method included interviews, observation and focused group discussions. The quantitative method employed questionnaires and secondary data analyses with multi variant test and Hotelling's T-square test.

## Results

The improvements were influenced by East Java Muhammadiyah culture, leadership and togetherness. This process was done step by step and was using role model in order to lessen the conflict that may have happened. On the other hand, in Central Java was much different. They realised but they did not respond to the condition. It was triggered by many factors, such as resignation, culture and leadership. There were many noticeable differences between the East and the Central, especially in their main activities and the support activities from Hospital Value Chain by Swayne. As a result the East Java Muhammadiyah hospitals showed noticeable differences comparing to the Central Java Muhammadiyah hospitals.

## Conclusions

This study concludes that the integrated management system of East Java Muhammadiyah Hospital is appropriate to be applied in all Muhammadiyah Hospital in Indonesia.

